# Genetic Variation in Telomere Maintenance Genes, Telomere Length and Breast Cancer Risk

**DOI:** 10.1371/journal.pone.0044308

**Published:** 2012-09-06

**Authors:** Jing Shen, Mary Beth Terry, Yuyan Liao, Irina Gurvich, Qiao Wang, Ruby T. Senie, Regina M. Santella

**Affiliations:** 1 Department of Environmental Health Sciences, Mailman School of Public Health, Columbia University, New York, New York, United States of America; 2 Department of Epidemiology, Mailman School of Public Health, Columbia University, New York, New York, United States of America; Tulane University Health Sciences Center, United States of America

## Abstract

**Background:**

Telomeres at the ends of eukaryotic chromosomes play a critical role in maintaining the integrity and stability of the genome and participate in the initiation of DNA damage/repair responses.

**Methods:**

We performed a case-control study to evaluate the role of three SNPs (*TERT-07*, *TERT-54* and *POT1-03*) in telomere maintenance genes previously found to be significantly associated with breast cancer risk. We used sister-sets obtained from the New York site of the Breast Cancer Family Registry (BCFR). Among the 313 sister-sets, there were 333 breast cancer cases and 409 unaffected sisters who were evaluated in the current study. We separately applied conditional logistic regression and generalized estimating equations (GEE) models to evaluate associations between the three SNPs and breast cancer risk within sister-sets. We examined the associations between genotype, covariates and telomere length among unaffected sisters using a GEE model.

**Results:**

We found no significant associations between the three SNPs in telomere maintenance genes and breast cancer risk by both conditional logistic regression and GEE models, nor were these SNPs significantly related to telomere length. Among unaffected sisters, shortened telomeres were statistically significantly correlated with never hormone replacement therapy (HRT) use. Increased duration of HRT use was significantly associated with reduced telomere length. The means of telomere length were 0.77 (SD = 0.35) for never HRT use, 0.67 (SD = 0.29) for HRT use <5yrs and 0.59 (SD = 0.24) for HRT use ≥5yrs after adjusting for age of blood donation and race and ethnicity.

**Conclusions:**

We found that exogenous hormonal exposure was inversely associated with telomere length. No significant associations between genetic variants and telomere length or breast cancer risk were observed. These findings provide initial evidence to understand hormonal exposure in the regulation of telomere length and breast cancer risk but need replication in prospective studies.

## Introduction

Telomeres are nucleotide repeats (TTAGGG) with relevant proteins at the ends of eukaryotic chromosomes, which play a critical role in maintaining the integrity and stability of the genome [1]; [2] Shortening of telomere length may gradually increase chromosome instability, and is correlated with susceptibility to bladder, esophageal, head and neck, lung, ovarian, renal cell and skin cancers (reviewed by Svenson et al.) [3]. The role of telomere length in breast cancer risk has been examined but with discrepant results. Two studies found significant correlations between longer telomeres and breast cancer risk [4]; [5], while others found non-significant correlations between shorter telomeres and breast cancer risk [6]–[10] or only significant associations in more aggressive subtypes (HER-2-positive and triple-negative tumors) [11]. Telomerase is a specialized reverse transcriptase that synthesizes the repeated telomere sequences, and positively regulates telomere length 12–14. Genetic variants in human telomerase reverse transcriptase (*TERT*), a catalytic subunit of telomerase, have been found to increase, decrease or have no association with breast cancer risk [15]–[18]. Telomere-binding proteins are critical for maintaining chromosome integrity and cellular function through regulation of telomere length and structure [19]; [20]. Deletions, mutations or polymorphisms in these genes might negatively regulate telomere length, cause dysfunction of telomere biology [21]; [22] and even increase cancer risk [18]; [23]; [24]. Using data from a large population-based case-control study, we previously identified three genetic variants (*TERT* rs2736109, rs3816659 and *POT1-03* rs33964002) in telomere maintenance genes associated with breast cancer risk [16], but no independent data has validated this finding.

In the current study, we evaluated the association between the three genetic variants and breast cancer risk among 313 sister-sets discordant for breast cancer from the New York site of the Breast Cancer Family Registry (BCFR). We also investigated the impact of these genetic variants as well as other epidemiologic factors on telomere length in unaffected sisters.

## Methods

### Study Subjects

The study population was selected from families participating in the New York site of the BCFR, six international collaborating sites. The description of the source of study participants and recruitment and data collection methods have been described in detail elsewhere [8]; [25]–[27]. Briefly, we recruited high-risk breast and/or ovarian cancer families from clinical and community settings within the metropolitan New York area starting in 1995. We selected sister-sets including at least one affected sister with breast cancer and at least one unaffected sister for the current study. We collected epidemiologic and family-history data including information on demographics, ethnicity, history of all cancers, smoking and alcohol consumption, reproductive history, hormone use, weight, height, and physical activity. A self-administered dietary questionnaire was also provided with return by mail. In addition, we collected a sample of peripheral blood from participants. The present study included a total of 313 breast cancer sister sets (333 breast cancer cases and 409 unaffected sisters). Three SNPs were genotyped among all 742 subjects, and telomere length was measured among 613 participants (278 cases and 335 unaffected sisters).

### Laboratory Methods

Genomic DNA was extracted from white blood cells (WBC) by the salting out procedure. WBCs were lysed with SDS (final concentration 0.66% SDS) in a nuclei lysis buffer and treated with RNaseA (final 150 µg/ml), RNaseT1 (final 20 U/ml) and Proteinase K (final concentration 150 µg/ml). Proteins were co-precipitation with NaCl (330 µl of saturated NaCl/1 ml of solution) by centrifugation. Genomic DNA was recovered from the supernatant by precipitation with 100% ethanol, washed in 70% ethanol and dissolved in TE buffer. Telomere length quantification was performed with the quantitative PCR (Q-PCR) method described by Cawthon [28] to determine the relative ratio of telomere (T) repeat copy number to a single-copy gene (S) copy number (T/S ratio) according to a 5-point standard curve using mixed human genomic DNA. This ratio is proportional to the average telomere length. Three SNPs were genotyped by TaqMan assays using a 7900 Real Time PCR system (Applied Biosystems, Foster City, CA) with allelic discrimination software supplied by the manufacturer. Ten percent of samples were re-assayed for genotyping after re-labeling to keep laboratory staff blinded, and the concordance was 100%.

### Statistical Analysis

Hardy-Weinberg Equilibrium (HWE) was tested to compare the observed and expected genotype frequencies among affected and unaffected sisters, respectively [29]. The more common allele of each of the three genetic variants in the telomere maintenance genes was selected as the reference group. Conditional logistic regression was used to model the association between the three SNPs and breast cancer risk within sister sets. The odds ratios (ORs) and corresponding 95% confidence intervals (CIs) were estimated by adjusting for age of blood donation. We also assessed the effect of the SNPs on breast cancer risk by using generalized estimating equations (GEE) to model the ORs [30], which allowed for sisters to be included even if they were concordant on genotype. We examined the association between the three SNPs and continuous telomere length among unaffected sisters using GEE linear models to account for the correlation among sisters [30]. We assessed the associations between telomere length and a series of covariates in unaffected sisters using a GEE model for linear regression analysis. The covariates included race (White, Hispanic, other), menopausal status (pre-, post-), BMI (<25, ≥25), smoking status (never, former, current), ever drink alcohol regularly (no, yes), hormone replacement therapy (HRT) use (never, ever), duration of HRT use (never, <5 yrs, ≥5yrs), oral contraceptive (OC) use (never, ever), duration of OC use (never, <5 yrs, ≥5yrs) and benign breast disease (never, ever). Any confounding variable that changed the correlation of telomere length and covariate by at least 10% was adjusted in the multivariable model. All analyses were performed with SAS software 9.2 (SAS Institute, Cary, NC).

## Results

Table 1 shows the characteristics of sisters in the New York site of the BCFR. Sisters who were affected with breast cancer were significantly older in age of blood donation (49 yrs) compared to their unaffected sisters (47 yrs) and more likely to be post-menopausal (64% vs. 34%) and former smokers (36% vs. 30%). Affected and unaffected sisters were similarly distributed with regard to ethnicity, BMI, ever alcohol drinker, HRT or OC use and history of benign breast disease.

**Table 1:** Characteristics of sisters in the New York site of the BCFR.

Characteristics		Affected	Unaffected	
		Sisters, N (%)	Sisters, N (%)	
Age at blood donation, Mean (SD)		49.2 (11.5)	47.4 (11.2)	
Race/Ethnicity	White	208 (63.0)	236 (57.8)	
	Hispanic	84 (25.5)	121 (29.7)	
	Other	38 (11.5)	51 (12.5)	
Menopausal status	Pre-	116 (35.6)	257 (66.1)	
	Post-	210 (64.4)	132 (33.9)	
BMI, Mean (SD)		25.5 (5.0)	25.5 (4.9)	
BMI status	<25	172 (52.1)	217 (53.7)	
	≥25	158 (47.9)	187 (46.3)	
Number of cigarettes per day*, Mean (SD)		13.9 (10.0)	13.0 (10.7)	
Smoking pack years*, Mean (SD)		13.3 (16.2)	10.9 (12.7)	
Smoking status	Never	196 (58.9)	235 (57.5)	
	Former	121 (36.3)	123 (30.1)	
	Current	16 (4.8)	51 (12.4)	
Ever drink alcohol	No	189 (57.1)	230 (56.4)	
	Yes	142 (42.9)	178 (43.6)	
HRT^#^ use	Never	281 (85.9)	322 (81.3)	
	Ever	46 (14.1)	74 (18.7)	
Duration of HRT^#^ use (years)		5.3 (7.1)	5.4 (7.3)	
OC use**	Never	126 (37.9)	158 (39.0)	
	Ever	206 (62.1)	247 (61.0)	
Duration of OC** use (years)		4.8 (4.6)	5.0 (4.5)	
Benign breast disease	Never	196 (60.9)	255 (64.6)	
	Ever	126 (39.1)	140 (35.4)	

* Among ever smokers;

#HRT: Hormone replacement therapy;

** OC: Oral contraceptive.

The distributions of *TERT-07*, *TERT-54* and *POT1-03* genotypes were compatible with those expected from the Hardy–Weinberg equilibrium. Overall, the three genetic variants in telomere maintenance genes had no significant impact on breast cancer risk after adjusting for age at blood donation (Table 2). The homozygous variant *AA* genotype of *TERT-07* was not associated with breast cancer risk (OR = 1.09, 95%CI: 0.56–2.12). The *TT* genotyping of *TERT-54* and *AA* genotyping of *POT1-03* were inversely associated with breast cancer risk but the associations were not statistically significant (the ORs were 0.83 (95%CI: 0.44–1.57) and 0.48 (95%CI: 0.23–1.00), respectively). The results obtained from the GEE models were consistent in magnitude with those from the conditional logistic regression models supporting an overall lack of association between these genotypes and breast cancer even after considering that sisters were more likely to be concordant on genotype.

**Table 2:** Breast cancer risk estimates by conditional logistic regression and GEE models in sister-sets for genotypes of telomere maintenance genes, New York site of the BCFR.

Genes	Genotypes	Affected Sisters, N (%)	Unaffected Sisters, N (%)	OR (95% CI)*	OR (95% CI)**
*TERT-07*	GG	146 (44.8)	179 (44.6)	1.00 (ref)	1.00 (ref)
rs2736109	GA	136 (41.7)	168 (41.9)	1.00 (0.66–1.51)	0.99 (0.77–1.29)
	AA	44 (13.5)	54 (13.5)	1.09 (0.56–2.12)	1.02 (0.71–1.45)
*TERT-54*	CC	135 40.5)	164 (40.2)	1.00 (ref)	1.00 (ref)
rs3816659	CT	150 (45.1)	183 (44.8)	1.00 (0.66–1.51)	1.01 (0.78–1.30)
	TT	48 (14.4)	61 (15.0)	0.83 (0.44–1.57)	0.94 (0.66–1.35)
*POT1-03*	GG	170 (51.5)	208 (51.6)	1.00 (ref)	1.00 (ref)
rs33964002	GA	136 (41.2)	152 (37.7)	1.06 (0.69–1.62)	1.13 (0.88–1.44)
	AA	24 (7.3)	43 (10.7)	0.48 (0.23–1.00)	0.67 (0.44–1.03)

* Conditional logistic regression model adjusted by age of blood donation;

** GEE model adjusted by age of blood donation.

Analyzing the associations between the subjects’ characteristics and telomere length within unaffected sisters (Table 3), we found that ever use of HRT and ever use of OC were significantly correlated with shorter telomere lengths compared with women who never used hormones after adjustment by age at blood donation. The β values of the GEE models were −0.11 (95%CI: −0.20–−0.02) and −0.09 (95%CI: −0.17–−0.01), respectively. Increased duration of HRT use was associated with shortened telomere length (0.77 for never use to 0.59 for HRT use ≥5yrs (*p* = 0.01)). The β value for HRT use ≥5 yrs was −0.16 (95%CI: −0.28–−0.03). Duration of OC use was also associated with significant shortening of telomere length (0.79 for never use and 0.71 for OC use <5 yrs, *p* = 0.03). The β value for OC use <5 yrs was −0.10 (95%CI: −0.19–−0.01). Ever use of hormones (HRT or OC) was associated with shorter telomere length for both premenopausal and postmenopausal women (data not shown); but only postmenopausal women with HRT use ≥5yrs had statistically significantly shorter telomeres (β = −0.13, 95%CI: −0.26–−0.01). Most of the observed significant associations were maintained after adjusting both for age, race and ethnicity, except for ever use of OC (Table 3).

**Table 3:** Association between characteristics of unaffected sisters (N = 335) and telomere length, New York site of the BCFR.

Subjects Characteristics		Unaffected	Telomere Length	GEE Model 1*		GEE Model 2**	
		Sisters	Mean (SD)	β	95%CI	β	95%CI
Age of blood donation		335	0.75 (0.34)	−0.003	−0.007–0.001	−0.002	−0.005–0.002
Ethnicity	White	216	0.69 (0.33)	0.00		0.00	
	Hispanic	82	0.82 (0.36)	0.13	0.02–0.23†	0.13	0.02–0.23†
	Other	36	0.92 (0.32)	0.23	0.13–0.34†	0.23	0.13–0.34†
Menopausal status	Pre-	214	0.77 (0.36)	0.00		0.00	
	Post-	104	0.72 (0.30)	−0.003	−0.11–0.10	−0.04	−0.15–0.06
BMI	<25	184	0.75 (0.35)	0.00		0.00	
	≥25	148	0.73 (0.33)	−0.02	−0.09–0.06	−0.03	−0.11–0.05
Smoking status	Never	192	0.77 (0.36)	0.00		0.00	
	Former	107	0.70 (0.30)	−0.06	−0.14–0.02	−0.01	−0.09–0.07
	Current	36	0.78 (0.35)	0.01	−0.12–0.13	0.03	−0.10–0.16
Ever drink alcohol	No	187	0.77 (0.35)	0.00		0.00	
	Yes	147	0.72 (0.33)	−0.05	−0.12–0.02	0.01	−0.06–0.08
HRT^#^ use	Never	269	0.77 (0.35)	0.00		0.00	
	Ever	56	0.64 (0.27)	−0.11	−0.20–−0.02†	−0.12	−0.21–−0.03†
Duration of HRT^#^ use (years)	Never	269	0.77 (0.35)	0.00		0.00	
	<5 yrs	35	0.67 (0.29)	−0.09	−0.20–0.02	−0.11	−0.22–−0.0002†
	≥5 yrs	20	0.59 (0.24)	−0.16	−0.28–−0.03†	−0.13	−0.25–−0.004†
OC^##^ use	Never	124	0.79 (0.35)	0.00		0.00	
	Ever	207	0.72 (0.33)	−0.09	−0.17–−0.01†	−0.06	−0.14–0.02
Duration of OC^##^ use (years)	Never	124	0.79 (0.35)	0.00		0.00	
	<5 yrs	122	0.71 (0.31)	−0.10	−0.19–−0.01†	−0.07	−0.16–0.02
	≥5 yrs	83	0.73 (0.36)	−0.08	−0.17–0.02	−0.05	−0.14–0.05
Benign breast disease	Never	207	0.74 (0.34)	0.00		0.00	
	Ever	116	0.73 (0.33)	−0.003	−0.08–0.07	0.02	−0.06–0.10

* adjusted by age of blood donation;

** adjusted by age of blood donation and ethnicity;

#HRT: Hormone replacement therapy;

##OC: Oral contraceptive;

† *p*<0.05.

Hispanic and other ethnic groups have significantly longer telomere lengths (0.82 and 0.92) compared with Caucasians (0.69). The β values of the GEE models were 0.13 (95%CI: 0.02–0.23) and 0.23 (95%CI: 0.13–0.34), respectively. No significant correlation was found between telomere length and other covariates within unaffected sisters (menopausal status, BMI, smoking status, ever drink alcohol and benign breast disease) (Table 3).

Investigating the associations between telomere length and genetic variants, we found no significant correlation between *POT1-03* and *TERT-54* variants and telomere length. We observed a significant inverse association for *TERT-07 GA* genotype and telomere length (Table 4).

**Table 4:** Association between genetic variants of telomere maintenance genes and telomere length in unaffected sisters, New York site of the BCFR.

Genes	Genotypes	Unaffected SisterN = 409	Telomere Length(Mean, SD)	GEE Model	
				β*	95%CI
*TERT-07*	GG	179	0.80 (0.35)	0.00	
rs2736109	GA	168	0.70 (0.31)	−0.10	−0.18–−0.01**
	AA	54	0.72 (0.40)	−0.10	−0.22–0.03
*TERT-54*	CC	164	0.76 (0.37)	0.00	
rs3816659	CT	183	0.73 (0.33)	−0.03	−0.11–0.06
	TT	61	0.76 (0.31)	−0.0004	−0.11–0.11
*POT1-03*	GG	208	0.71 (0.33)	0.00	
rs33964002	GA	152	0.78 (0.34)	0.06	−0.02–0.15
	AA	43	0.75 (0.35)	0.05	−0.08–0.18

* adjusted by age of blood donation;

** *p*<0.05.

## Discussion

Recently, increasing evidence suggests that telomere maintenance genes, such as *TERT*, *TERC*, *TERF1*, *TERF2*, *TINF2*, *TERF2IP* and *POT1* are associated with cancer risk [15]–[18]; [21]–[24]. SNPs in three genomic regions including the *TERT*-*CLPTM1L* locus at 5p15.33, a 181 kb linkage disequilibrium (LD) block at 12p13 and a region at 14q21 were also correlated with multiple cancer risks [31]–[33]. These genomic regions influence cancer risk through regulating the telomere pathway. Variant rs401681 in the *TERT*-*CLPTM1L* locus might lead to gradual shortening of telomeres over time [31], but another study did not find significant associations of this SNP with telomere length or the risks of melanoma, breast and colorectal cancer [34]. The variant alleles of four SNPs in the 14q21 region were associated with longer telomere length, and one SNP was significantly associated with longer telomeres and reduced risk of bladder cancer [32]. The locus at 12p13 was associated with telomerase activity [33] probably due to its containing *ATF7IP*, a gene that enables expression of *TERT* and regulates the RNA component encoded by *TERC* [35]. We previously identified three genetic variants in telomere maintenance genes (*TERT* and *POT1*) that were significantly correlated with breast cancer risk in a large (1,067 cases, 1,110 controls) case-control study, the Long Island Breast Cancer Study Project [16]. Here, the same three SNPs were investigated among 333 cases and 409 unaffected sisters. We were unable to replicate the results in LIBCSP, i.e. no significant association was observed between those genetic variants and breast cancer risk, nor were they associated with telomere length. The inconsistent results on the role of telomere genetic variants may be a result of the complex nature of telomere homeostasis, differences in genetic backgrounds for the study populations and the overall sample sizes.

Another recent study found that a SNP near *TERC* was significantly associated with shorter telomere length, but this result could not be replicated in a family-based study possibly due to its minor effect, which raises another issue, statistical power [36]. We had a small overall sample size, which limited the statistical power of this study. Even if a genetic variant in a telomere pathway gene impacts telomere length phenotype and thus breast cancer risk, the relatively weak effect may be insufficient to result in phenotypic alterations.

In addition, long-term environmental exposures and gene-environment interactions may also contribute to the observed inconsistencies. Several studies have shown that environmental risk factors like cigarette smoking [37]; [38], obesity [37]; [39]; [40], oxidative stress [41], less physical activity [42], inflammation [43], traffic pollution [44] and polycyclic aromatic hydrocarbon exposure [45] were significantly correlated with short telomere length, but there is no conclusive data on the role of hormone exposure. In the current study, ever use of HRT or OC was statistically significantly associated with shortened telomere length. With increased duration of HRT use (≥5 yrs), telomere length was significantly decreased (from 0.77 for never use to 0.59 for HRT use ≥5 yrs). A similar association between ever hormone use and short telomere length was observed for premenopausal and postmenopausal women. These findings are consistent with a few but not all previous studies. In a sister study with 647 women, no significant correlation was observed between use of hormone replacement therapy and telomere length [46]. A study conducted in an Asian population (130 women) found that postmenopausal women who had been on hormone therapy for more than five years had longer telomeres compared to age-matched women who had no hormone therapy [47]. However, telomere length among hormone users may reflect other health behaviors previously associated with longer telomeres, such as regular exercise and daily vitamin supplements [42]; [48]; the correlation between hormone therapy and telomere length may thus have been confounded. Investigations using large sample sizes are warranted to clarify the actual association between telomere length and hormone use. Another study that included 53 postmenopausal women found differing associations between endogenous and exogenous estrogen exposures and telomere length. Endogenous estrogen exposure (length of reproductive years of life as a proxy) was significantly associated with longer telomere length (β = 0.06, *p* = 0.04) while longer duration of exogenous hormone use was linked to shorter telomere length in a subgroup of 18 women with surgical menopause (β = −0.11, *p* = 0.02) [49]. No significant effect was observed for hormone use on telomere length among women with natural menopause [49]. Another recent study found that exposure to prenatal stress was significantly associated with shorter telomere length in young adulthood, especially for women (*p*<0.01) [50]. These results suggest a mechanism of shortened telomere length through transplacental transport of the hormone cortisol and maternal release of placental hormones.

One strength of the current study is the family-based design that efficiently reduces potential confounding related to population admixture [51]. The confounding effects due to differences in genetic susceptibility as well as behavioral and lifestyle factors that cluster within families may be reduced through this design. A number of limitations need to be addressed. First, we have no phenotype data for the different genetic variants, which limits our ability to establish a direct biological connection between genetic polymorphisms and telomere length and breast cancer risk. In contrast to previous studies [37]–[40]; [52], factors such as age, BMI and smoking were not associated with telomere length in the current study. This could be due to the small sample size, which also did not allow us to explore potential gene-environmental interactions and modification by covariates. In addition, since telomere length is largely genetically determined [53]; [54], the sibling design may be too closely matched and dilute the moderate associations between genetic polymorphisms and breast cancer risk.

In conclusion, we found that exogenous hormonal exposure was inversely correlated with telomere length, but genetic variants were not associated with telomere length or breast cancer risk. Further prospective investigations focused on various hormonal exposures and telomere alterations may help in our understanding the role of these factors in regulating breast cancer risk.
